# Introducing Preference Epidemiology: Improving Patient-Centered Approaches in Health Decision-Making

**DOI:** 10.3389/ijph.2025.1608617

**Published:** 2025-05-15

**Authors:** Giovanni Spitale, Federico Germani, Nikola Biller-Andorno

**Affiliations:** ^1^ Institute of Biomedical Ethics and History of Medicine, University of Zurich, Zurich, Switzerland; ^2^ Center for Medical Ethics, University of Oslo, Oslo, Norway

**Keywords:** preference epidemiology, patient-centered care, health policy, health literacy, health communication strategies

Two pivotal events in 2024 – the USPSTF (United States Preventive Services Task Force)’s revised recommendations for cancer screening, and the symbolic “end of an era” for Dartmouth Atlas marked by the passing of Jack Wennberg, alongside shifts in the Center for Medicare and Medicaid Services’ policies on claims data access – underscore the urgent need to reflect on the integration of patient preferences into healthcare decision-making and policy development. Healthcare decisions often demand that individuals weigh the potential benefits of an intervention against possible burdens, risks and harms, a process influenced by personal values, beliefs, and prior experiences. Traditionally, public health policies have focused on population-level evidence on key performance indicators of healthcare practices (such as the number of deaths prevented by such practices) to guide recommendations. However, this approach can overlook the nuanced ways in which individuals process information and make choices, which are not always driven solely by the potential of a healthcare practice to reduce mortality risk. The Dartmouth Atlas has already demonstrated how healthcare practices and outcomes can vary significantly across regions, depending on factors such as practice patterns, physician preferences, resources availability, and patient demographics [1]. Preference epidemiology offers a framework for understanding how individuals value trade-offs between benefits and burdens, risks, and harms of a certain healthcare practice, paving the way for a more patient-centered approach to healthcare [2]. Thus, preference epidemiology can determine how benefits and burdens, risks and harms are weighed by people.

Patient decisions on medical treatment are rarely straightforward. Different individuals may understand or respond to the same information in diverse ways, depending on their unique thresholds for risk tolerance, burden or harm acceptance (for themselves or for their families), and their prioritization of benefits, as exemplified in the case of PSA (Prostate Specific Antigen) screening for prostate cancer detection [3–5]. People’s values and past experiences can deeply shape their health choices: for instance, in the case of PSA screening, individuals with a family history of illness may be more inclined to pursue screening tests, while those with previous negative healthcare experiences might hesitate [2, 4, 5]. Preference epidemiology systematically investigates these factors, shedding light on why individuals may choose certain healthcare options over others. In a similar way, the history of mammography recommendations for breast cancer detection highlights the challenges of communicating nuanced risk-benefit information to the public. Although graded as beneficial by the United States Preventive Services Task Force (USPSTF) in 2002, the recommendation was later modified multiple times (most recently in June 2024) [6–9], sparking confusion in the US and abroad. A primary concern here is that these recommendations for the integration or use of healthcare practices are based on assumptions regarding their worthiness, without knowing if those concerned–the patients and their families – would have similarly weighed the benefits, risks, burdens, and potential harms of such practices. These aspects would be considered by preference epidemiology research, leading to tailored, evidence-based recommendations and communication that integrate clinical evidence with evidence on patients’ diverse values, perceptions, and preferences [8, 10].

In the case of screening practices, some patients may value greatly the benefit of an early detection of disease, despite a risk of overdiagnosis or overtreatment, while others may prefer to avoid risking any unnecessary intervention, even at the cost of a delayed diagnosis. Preference epidemiology aims to capture these varied viewpoints, systematically identifying the specific conditions under which individuals find particular interventions acceptable or not. A core goal of preference epidemiology is to determine the thresholds at which individuals find a medical intervention acceptable [2, 11], provided that these decisions are informed and autonomous – guaranteed by sufficient medical and information literacy. These insights allow healthcare systems to tailor their programs, policies, and communication strategies to better align with the values of target populations, enhancing the population’s ability to make informed and autonomous choices, and enhancing the relevance and impact of healthcare offerings.

Preference epidemiology is significant both at the population and individual level: when policymakers understand the acceptability thresholds of different segments of the population, they can design healthcare programs that better resonate with those groups. This alignment increases both the effectiveness and uptake of healthcare services, benefiting individuals and public health as a whole [2]. Targeted communication is essential for translating medical information into messages that diverse individuals can relate to and understand [2, 12], and by identifying how people evaluate trade-offs, preference epidemiology can help shape communication that enhance health literacy and promote informed decision-making. A cornerstone of preference epidemiology is therefore to promote open, transparent dialogue between patients and healthcare providers. When providers understand a patient’s perceptions, values, and preferences, they can offer personalized guidance that supports patients in making decisions aligned with both evidence and their own priorities [4, 5, 13–15].

The principles of preference epidemiology are applicable across a range of health interventions that require complex decision-making involving trade-offs based on benefits, burdens, risks and harms. This approach emphasizes the importance of aligning healthcare services with the informed and autonomous preferences of the population. This approach can guide patient-centered policies for interventions, e.g., mammography, colonoscopy, or hypertension screening in adolescents. PSA screening has been a focal point for our initial exploration of preference epidemiology due to the complex interplay of factors that influence individual decision-making surrounding this screening test. Additionally, its use at the population level remains controversial due to conflicting recommendations and an uncertain benefit-to-harm ratio [16–20]. Studies show that individuals often overestimate the benefits of PSA screening, reflecting a gap between perception of PSA screening and clinical data to support its use at the population level [2, 4, 5, 13, 14]. This discrepancy is particularly evident among individuals with personal or family experiences with cancer: for instance, men with a family history of prostate cancer may favor PSA screening despite the risks of overdiagnosis, prioritizing peace of mind over clinical probabilities [2, 13]. In contexts like this, preference epidemiology data can inform policies that not only rely on clinical evidence, but also respect and address patients’ concerns and expectations, allowing for health policy decisions that consider citizens’ values, preferences, priorities, and enabling the development of more responsive healthcare communication strategies.

Preference epidemiology represents a largely untapped resource and a shift toward understanding the subjective dimensions of healthcare decision-making. By emphasizing the role of individual values, preferences, and experiences, it enables the development of healthcare policies and communication strategies that are both evidence-based and patient-centered. Methodologically, preference epidemiology must balance quantitative rigor with qualitative depth: it should be quantitative enough to rapidly gather evidence that supports robust, actionable inferences, while remaining qualitative enough to capture the deeper meanings and motivations behind individuals’ values and choices. As the field advances, its application across diverse health contexts has the tremendous potential to improve health literacy and empower individuals in making informed, autonomous decisions. By setting the agenda and defining the role of preference epidemiology, we aim to help healthcare systems align more closely with the populations they serve, promoting a more responsive and effective public health landscape.
